# Robust and Fast Point Cloud Registration for Robot Localization Based on DBSCAN Clustering and Adaptive Segmentation

**DOI:** 10.3390/s24247889

**Published:** 2024-12-10

**Authors:** Haibin Liu, Yanglei Tang, Huanjie Wang

**Affiliations:** 1 College of Mechanical and Energy Engineering, Beijing University of Technology, Beijing 100124, China; liuhb@bjut.edu.cn; 2 Shanghai Spaceflight Precision Machinery Institute, Shanghai 201600, China; yangleibll@163.com

**Keywords:** robot localization, point cloud registration, normal distribution transform (NDT), density-based spatial clustering of applications with noise (DBSCAN), clustering and segmentation

## Abstract

This paper proposes a registration approach rooted in point cloud clustering and segmentation, named Clustering and Segmentation Normal Distribution Transform (CSNDT), with the aim of improving the scope and efficiency of point cloud registration. Traditional Normal Distribution Transform (NDT) algorithms face challenges during their initialization phase, leading to the loss of local feature information and erroneous mapping. To address these limitations, this paper proposes a method of adaptive cell partitioning. Firstly, a judgment mechanism is incorporated into the DBSCAN algorithm. This mechanism is based on the standard deviation and correlation coefficient of point cloud clusters. It improves the algorithm’s adaptive clustering capabilities. Secondly, the point cloud is partitioned into straight-line point cloud clusters, with each cluster generating adaptive grid cells. These adaptive cells extend the range of point cloud registration. This boosts the algorithm’s robustness and provides an initial value for subsequent optimization. Lastly, cell segmentation is performed, where the number of segments is determined by the lengths of the adaptively generated cells, thereby improving registration accuracy. The proposed CSNDT algorithm demonstrates superior robustness, precision, and matching efficiency compared to classical point cloud registration methods such as the Iterative Closest Point (ICP) algorithm and the NDT algorithm.

## 1. Introduction

Robot localization plays a critical role in achieving the autonomy, reliability, and efficiency of robots, which are essential for their widespread applications across various domains. The primary methods for robot localization encompass technologies such as Global Positioning Systems (GPS) [1], Inertial Navigation Systems (INS), visual sensors [2], and LiDAR (Light Detection and Ranging) [3]. Traditional GPS exhibits certain deficiencies in providing attitude estimation owing to issues such as multipath effects and delay, thereby limiting its application within indoor environments [4]. In recent years, extensive research has been conducted in the realm of attitude estimation based on Inertial Navigation Systems (INS) and visual sensors. INS employs the integration of acceleration and angular velocity to estimate attitude information. However, the presence of biases and noise in inertial sensors result in the predicament of accumulated error in estimation [5]. Visual sensors offer robust and accurate motion estimation, but they remain susceptible to the influence of ambient lighting conditions [6]. In contrast, LiDAR, as an active sensor, measures obstacles in the environment by emitting laser pulses and recording the time taken for the reflected pulses to return. This generates detailed point cloud data that can be used to construct high-precision maps of the environment, aiding robots in tasks such as localization, navigation, and obstacle avoidance [7,8]. With its high accuracy, stability, and long-range sensing capabilities, LiDAR has established itself as a key technology in fields such as autonomous driving, unmanned aerial vehicles, and robotics.

LiDAR-based localization algorithms analyze and process continuous point cloud data to provide real-time position information and environmental modeling for robots or vehicles. These algorithms ensure autonomous navigation in complex environments. Point cloud registration (often referred to as scan matching) is one of the core steps in achieving robot localization and navigation. Its main task is to accurately align point clouds acquired at different times or locations, mapping them to a unified coordinate system to extract the robot’s trajectory information and environmental details.

In the actual process of point cloud registration, the first step is to establish associations between points or cells in consecutive LiDAR scan frames. This involves identifying corresponding elements between two sets of point clouds. Then, a cost function is constructed to describe the error or matching quality between the point clouds. Common cost functions include minimizing the distance between points, the distance between feature points, or the similarity between distributions. Finally, optimization techniques, such as gradient descent, least squares, or advanced nonlinear optimization methods, are used to estimate the relative pose transformation matrix between the point clouds. Through these steps, the robot can continuously update its precise position within the environment, ensuring precise localization for autonomous navigation and effective obstacle avoidance.

In the research and application of LiDAR point cloud registration, registration methods are generally divided into three categories: point-based registration, feature-based registration, and distribution-based registration.

Point-based registration methods directly utilize the raw point data in the point cloud, aligning the point clouds by identifying corresponding points between two sets of point clouds and minimizing their Euclidean distance or other geometric errors. The classic Iterative Closest Point (ICP) algorithm [9] is a representative of this approach. ICP iteratively identifies the closest point pairs and calculates the rigid transformation between the point clouds. However, ICP assumes that the closest point pairs are always correctly matched, an assumption that often fails in real-world applications. In complex environments, the presence of noise, outliers, or sparse data can significantly reduce the algorithm’s accuracy. Additionally, ICP’s computational cost is high due to the exhaustive search for point correspondences, making it less efficient when processing large-scale point clouds.

Unlike point-based methods, feature-based registration methods perform registration by extracting distinctive geometric features from the point cloud, such as corners, edges, and planes [10,11]. These methods are more robust when dealing with sparse point clouds or occlusions compared to directly processing all points. Even in scenarios with missing or incomplete data, feature points can still facilitate effective alignment. However, the success of feature-based methods heavily depends on the precision of feature extraction, as inaccuracies in the extracted features can significantly affect the final registration outcome. These methods are particularly well-suited for environments with stable geometric characteristics and can deliver reliable matching performance across varying viewpoints.

Distribution-based registration methods treat the point cloud as a probability distribution in continuous space and use probability density functions for alignment. The Normal Distributions Transform (NDT) [12] is a typical representative of this approach. NDT divides the point cloud into a regular grid of cells and uses normal distributions to describe the distribution of points within each cell. Compared to ICP, NDT demonstrates faster registration speed and greater robustness when handling large-scale scenes, particularly in large-scale environments with noise or extensive point cloud data [13]. However, NDT may introduce discontinuities at the boundaries of the grid cells.

The size of the cells is predefined by the user and typically selected based on application requirements and environmental characteristics. Determining the optimal cell size requires estimating data through multiple experiments to achieve relatively superior registration results. The size of the cells determines the resolution of the NDT model. When the cell size is set too large, it fails to reflect the features of the point cloud accurately. Conversely, overly small cell sizes make the algorithm more vulnerable to noise from LiDAR scanning equipment, which can degrade performance. Furthermore, point cloud data collected in different environments require different optimal cell resolutions. In some cases, insufficient data within a grid cell can prevent the calculation of a reliable Gaussian distribution. Therefore, grid resolution is a crucial parameter that directly affects the algorithm’s accuracy. To address the challenge of determining optimal cell parameters, researchers have proposed multi-resolution NDT. While this approach enhances adaptability, it also substantially increases the computational complexity and load of the algorithm in practical applications.

Moreover, traditional cell-based methods often struggle to extract critical features from the original point cloud data, especially at the intersections of two planes where the normal vector of the point cloud undergoes abrupt changes. Due to the uniform cell division in point cloud space, the point cloud at the intersection is likely to be assigned to the same cell block. However, features such as the cell mean or covariance extracted through traditional NDT may not adequately describe the point cloud features at these locations, which can significantly affect the accuracy of point cloud matching algorithms. Therefore, the selection and division of cell size play a vital role in the accuracy and robustness of the NDT algorithm.

To address the challenges of irrational cell subdivision and distortion in NDT, this paper proposes a novel registration algorithm based on cluster segmentation. It integrates considerations of point cloud shape, segmenting the reference frame’s point cloud into linear clusters. Adaptive cell partitioning is executed based on point cloud size. The improved Density-Based Spatial Clustering of Applications with Noise (DBSCAN) algorithm is incorporated for denoising and clustering the reference frame’s point cloud. Singular Value Decomposition (SVD) is also employed to partition the point cloud into linear shapes, followed by the determination of cell lengths based on point cloud size. To ensure finer granularity, excessively large cells are further subdivided into overlapping smaller cells. Ultimately, optimization of the objective function for all matched items is achieved through Gaussian distribution functions resulting from the dual cell partitioning. Compared to classical registration algorithms like ICP and NDT, the proposed method demonstrates superior robustness, precision, and matching efficiency.

In summary, the primary contributions of this paper encompass the following aspects:

(1) An innovative method is introduced for adaptively setting DBSCAN parameters based on local point cloud density. By calculating the average nearest-neighbor distance, it enhances the clustering’s robustness and adaptability across a wide range of environmental conditions.

(2) An innovative approach based on density clustering for local point cloud feature clustering is proposed. This approach facilitates the segmentation of point clouds into linear clusters and thereby establishes a foundation for the generation of adaptive grid cells within the algorithm.

(3) A novel strategy for NDT cell partitioning is proposed. In this method, NDT cells are adaptively generated based on the size of linear point cloud clusters, and the decision to continue dividing is adjusted by the cell’s length. This two-layer cell structure serves distinct roles in the optimization phase: the first layer of cells enhances the algorithm’s matching range, while the second layer improves its accuracy.

The structure of this paper is as follows: Section 2 reviews related works on NDT-based point cloud registration. Section 3 elucidates the fundamental process of NDT, highlighting the deficiencies in NDT algorithms. Section 4 expounds extensively on the proposed approach. Section 5 demonstrates the experimental comparison results. Section 6 gives the discussion. Lastly, Section 7 summarizes the contents of this paper.

## 2. Related Work

The NDT methodology was first introduced by Bieber et al. in 2003, and extended to three dimensions by Martin Magnusson in 2009 [14]. NDT utilizes lidar scan points as input and matches them to target points through potential probability conditions. Typically, reference point clouds are subdivided into uniformly sized cells, a step referred to as cellization. Subsequently, the mean and covariance of each cell are computed, and an optimization objective is solved for each scan point and its corresponding cell. The computational efficiency of NDT is closely tied to the number of target cells, which can be controlled by adjusting the size of the subdivided cells. The original approach can also be viewed as a point-to-distribution (P2D) method, as it directly matches scan points with the probabilistic distribution of unitized objectives. Later, Stoyanov applied probabilistic methods to scan and target points to enhance registration speed [15].

The cell partitioning within the NDT algorithm stands as a pivotal step impacting robustness, matching precision, and operational efficiency. Researchers have made various enhancements to this step. Cihan et al. developed the ML-NDT algorithm [16], which substitutes a leave function for the Gaussian probability density function as the scoring function and optimizes the score function using Newton and Levenberg–Marquardt methods. This approach divides point clouds into 8n cells (where n represents the number of layers) to enhance matching precision but concurrently increases computational complexity. Das et al. proposed the multi-scale k-means NDT (MSkM-NDT) approach [17], addressing the discontinuity issue in the NDT cost function through multi-scale optimization and k-means-based point cloud segmentation. However, multi-scale optimization prolongs convergence time, and k-means-based point cloud segmentation struggles with accuracy when cluster numbers are unknown. Additionally, the scholars introduced the Segmented Region Growing NDT (SRG-NDT) approach [18], first removing the ground plane and then using a region-growing algorithm for clustering the remaining points to enhance computational speed. Lu et al. proposed a variable cell size NDT algorithm [19], aiming to improve accuracy but demonstrating limited effectiveness with sparse point clouds. Liu et al. introduced the Composite Clustering NDT (CCNDT) method [20], which employs clustering points to calculate probability distributions and employs DBSCAN and k-means clustering algorithms for grid partitioning, replacing constant grid size. However, for complex point clouds, this method struggles to maintain the continuity and local features of objective points. Hong et al. tackled the issue of discontinuity caused by the discretization of regular cells in NDT by introducing an interpolation method based on overlapping regular cells. They applied this method to point-to-distribution NDT registration (NDT-P2D) [21].

In addition to refining grid cell partitioning, researchers have explored integrating other information into the pose estimation process to enhance matching accuracy. Andreasson et al. extended cost constraints by incorporating pose information into the NDT-D2D (Distribution to Distribution) method [22]. Liu et al. proposed an improved NDT algorithm, termed INDT, which employs only pre-processed feature points for matching [23]. This method employs Fast Point Feature Histogram (FPFH) descriptors and the Hausdorff distance method to extract feature points and enhance precision using a hybrid Probability Density Function (PDF). ParkG et al. introduced a novel pose estimation scheme [24], wherein vertices and corners are extracted from 2D lidar scan point clouds within the NDT framework, enhancing efficiency and performance. Shi et al. introduced an algorithm called NDT-ICP, sequentially combining and operating NDT and ICP. This bifurcates the matching process into two stages: NDT for coarse registration, followed by ICP for fine alignment. This hybrid mechanism significantly improves NDT’s registration performance [25,26]. Chiang et al. addressed the initialization issue of NDT by proposing two strategies: combining an inertial navigation system with a global navigation satellite system, and processing point clouds in each partitioned scanning area based on density ratios [27].

Currently, researchers typically prioritize precision over operational efficiency when performing cell subdividing within the NDT algorithm. The approach generally involves point cloud segmentation, which is effective but necessitates precise segmentation of point clouds, leaving room for improvement. Furthermore, while enhancing matching accuracy through the addition of extra information is feasible, it inevitably increases the computational burden. Therefore, to address the current research gaps, this paper aims to achieve accurate point cloud registration results swiftly and robustly.

## 3. Preliminaries

The conventional NDT algorithm can be delineated into three primary steps. Initially, the reference point cloud undergoes uniform subdividing into equal-sized cells $C_{i=1\ldots n}$. If the cell $C_{i=1\ldots n}$ contains more than two points, the following procedures are carried out:

(1) Identification of points $m_{i}$ within each grid cell $C_{i=1\ldots n}$.

(2) Calculate the mean μ of points within each cell as follows:

(1) $$\mu =\frac{1}{n}\sum_{i=1}^{n}m_{i}$$

(3) Compute the covariance matrix for the points within each cell as follows:

(2) $$\sum =\frac{1}{n}\sum_{i=1}^{n}(m_{i}-\mu ){\left(m_{i}-\mu \right)}^{T}$$

The Gaussian distribution N(μ,∑) can model the distribution of points within a grid cell, and the Probability Density Function (PDF) $p(x_{i})$ is represented by

(3) $$p(x_{i})\sim \exp(-\frac{{\left(x_{i}-\mu \right)}^{T}\sum^{-1}(x_{i}-\mu )}{2})$$

where $x_{i}$ represents a point within the current scan C and $p(x_{i})$ signifies the probability of $x_{i}$ being contained within a cell characterized by a Gaussian distribution.

**Figure 1:**
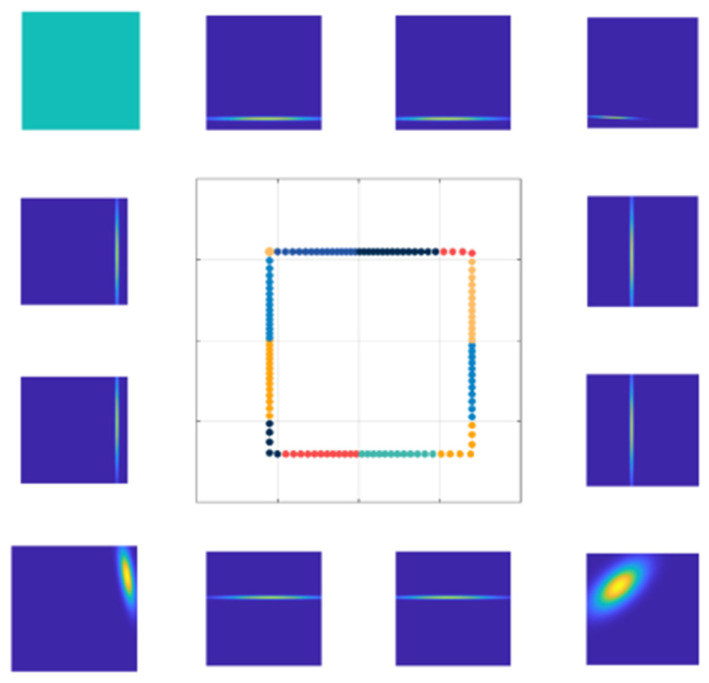
NDT map which visualizes the PDF within each grid cell.

Similar to an occupancy grid, NDT establishes a grid of regular subdivisions. However, in contrast to occupancy grids that denote the probability of cell occupancy, NDT’s cell grid signifies the probability distribution of point clouds within each individual cell. Typically, a cell grid of dimensions 1 m × 1 m is conventionally adopted. This approach describes a plane in a segmented, continuous, and differentiable manner using the form of probability density. Figure 1 visualizes the PDF within each grid cell, commonly referred to as the NDT map.

The subsequent step following grid partitioning is the registration process, which proceeds as follows:

(1) Construct the NDT map of the reference point cloud.

(2) Initiate parameter estimates, which can be initialized with zero values or odometry data.

(3) Transform each point $x_{i}$ using the transformation matrix p to obtain the corresponding mapped point $x_{i}^{\prime }$, as described by Equation (5).

(4) Identify the corresponding Gaussian distribution grid cell for each mapped point $x_{i}^{\prime }$.

(5) Calculate the score for each mapped point $x_{i}^{\prime }$ by computing their scores and summing the results, determining the score $S(x_{i}^{\prime })$ for the parameters, as outlined in Equations (6) and (7).

(6) Iterate to calculate a new parameter estimate by attempting to optimize the obtained scores.

(7) Return to step (3) and iterate through the process until the convergence criterion is satisfied.

This stepwise procedure illustrates the sequence of operations involved in the registration process, ultimately aligning the current scan with the reference point cloud using the NDT framework.

(4) $$p=[t_{x},t_{y},\phi ]$$

(5) $$T(p,x_{i})=\left(\begin{array}{cc} \cos\phi & -\sin\phi \\ \sin\phi & \cos\phi \end{array}\right)x_{i}+\left(\begin{array}{c} t_{x}\\ t_{y} \end{array}\right)$$

where p signifies the transformation matrix, $t_{x},t_{y}$ elucidates translation, and ϕ delineates the inter-frame rotation.

(6) $$p(x_{i}^{\prime })=\exp(-\frac{{\left(x_{i}^{\prime }-\mu \right)}^{T}\sum^{-1}\left(x_{i}^{\prime }-\mu \right)}{2})$$

(7) $$S(x_{i}^{\prime })=-\sum_{k=1}^{n}P(x_{i}^{\prime })$$

The final step involves optimizing the score $S(x_{i}^{\prime })$ by calculating the gradient g and the Hessian matrix H of $S(x_{i}^{\prime })$. An optimization algorithm is employed to enhance the transformation parameter p, solving the following equation iteratively at each iteration as follows:

(8) HΔp=−g

(9) $$g_{i}=\frac{\partial f}{\partial p_{i}}$$

(10) $$H_{ij}=\frac{\partial f}{\partial p_{i}\partial p_{j}}$$

Compared to the ICP algorithm, the NDT algorithm demonstrates enhanced robustness and lower computational demands. These advantages stem from the NDT algorithm’s utilization of a set of local Gaussian distributions to model the distribution of scanned point clouds. Traditional NDT algorithms subdivide the initial scan into evenly sized grid cells in a regular manner and employ four overlapping grids to minimize the effects of discretization, allowing for an accurate representation of the point cloud distribution within the initial scan. However, directly partitioning point cloud data into uniformly sized and closely connected cells without considering the actual shape of the point cloud can obscure the local distribution features of the point cloud. Consequently, the NDT algorithm faces challenges in adapting to abrupt variations in local point cloud distributions. Its accuracy is also limited by the fixed cell size, especially near corners and gaps in the point cloud. Figure 1 illustrates an example of an NDT map, with different point clouds within distinct cells depicted in various colors. The outermost shape in the figure is an enlarged version of the NDT map, which displays a visualization of the probability density function of the point cloud within each cell. A higher probability density indicates brighter, denser portions of the observed point clouds. From the figure, it is apparent that the probability density distribution within grid cells containing corner points is less focused, indicating the NDT map’s inability to precisely capture the shape features of the point cloud at those locations. Additionally, the top-left cell is discarded due to the scarcity of points, rendering it unable to form an NDT map and leading to the loss of local point cloud information. Moreover, considering the use of four overlapping grids, representing a 2.5 m × 2.5 m point cloud as an NDT map requires 48 1 m × 1 m grid cells, thereby increasing the overhead in terms of storage space and optimization time.

**Figure 2:**
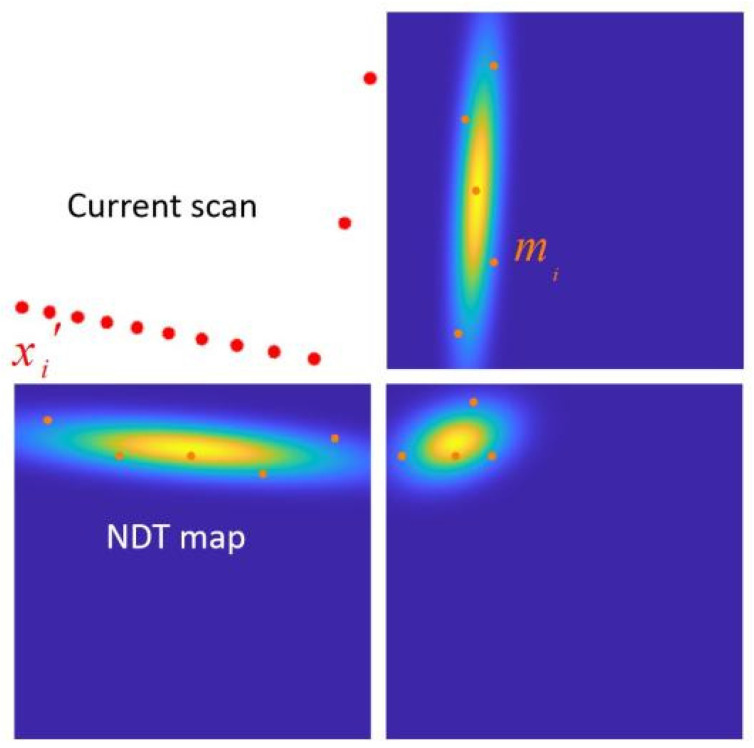
Illustration of the current point cloud being mapped to the NDT map.

A fixed grid partitioning not only diminishes the algorithm’s precision but also impacts its robustness. In the fourth step of the NDT algorithm, determining the corresponding Gaussian distribution grid cell for each mapped point $x_{i}^{\prime }$, most mapped points can accurately fall within their respective grid cells when the positions of the two-point cloud frames are similar. However, in real-world applications, point cloud frames often differ significantly in position and shape. This discrepancy can result in mapped points falling outside the boundaries of the NDT map or being erroneously assigned to incorrect grid cells. This situation can lead the algorithm into local optima, resulting in an incorrect estimation of the transformation matrix. While increasing the size of grid cells can alleviate this problem, larger grid cells, when transformed into Gaussian distributions, may lose more local distribution characteristics. Figure 2 illustrates a scenario where the current point cloud is mapped to the NDT map. The red points represent mapped points $x_{i}^{\prime }$, while the NDT map is formed by the orange reference point cloud $m_{i}$. Several mapped points fall into blank areas, where they are effectively discarded, contributing no useful information to the subsequent optimization process. This phenomenon underscores the challenge of balancing grid size for accurate mapping and capturing local distribution details within the NDT algorithm.

## 4. Method

To overcome the challenges of distortion and erroneous mapping in NDT maps caused by fixed cell partitioning, this paper introduces a novel approach for grid cell subdivision. This method optimizes the use of grid cells, requiring fewer subdivisions while offering a more accurate representation of the point cloud distribution. By doing so, it significantly enhances the accuracy, robustness, and operational efficiency of point cloud registration. Compared to traditional methods employing quadruple-overlapping grids, the proposed approach notably reduces computational complexity.

### 4.1. Point Cloud Clustering and Segmentation

This work initiates with an enhancement of the DBSCAN clustering algorithm, incorporating a discriminative mechanism during the cluster expansion phase to acquire linear point cloud clusters. The improved algorithm is referred to as L-DBSCAN. It is applied to cluster the input reference scans R, facilitating the segmentation of point clouds into linear point cloud clusters C and thereby providing a precise foundation for subsequent cell generation.

DBSCAN, a density-based spatial clustering algorithm, proves instrumental in identifying clusters of arbitrary shapes within spatial databases that may contain noise. The algorithm proceeds as follows:

(1) Begin by arbitrarily selecting a data point and identifying all data points within a distance of eps from this point. If the count of these data points is less than minpts (a specified numerical threshold), label the point as noise. If the count is greater than or equal to minpts, mark the point as a core sample and assign a new cluster label $C_{n}$.
(2) Proceed to visit all neighbors of the core sample within a distance of *eps*. If a neighbor has not been assigned a cluster label, allocate it to $C_{n}$ and continue expanding the cluster. If the neighbor is also a core sample, recursively visit its neighbors until no more core samples are within the *eps* distance.
(3) Select another unvisited data point and repeat the above steps until all data points have been visited.

The DBSCAN algorithm relies on two critical parameters: the neighborhood radius (eps) and the specified minimum number of samples (minpts). The selection of these parameters significantly influences the clustering outcome. A smaller value of eps will lead to the discovery of more clusters, whereas a larger value of minpts will require a greater number of core samples to form a cluster. Through this density-based clustering approach, DBSCAN exhibits flexibility in identifying clusters of arbitrary shapes and demonstrates a certain degree of robustness when dealing with noisy data.

Generally, the parameters of the DBSCAN algorithm, such as the neighborhood radius and minimum sample size, are typically adjusted empirically. However, these empirical rules often fail to adapt to the variations in different point cloud datasets, especially when the datasets have varying densities or complex geometric features. Improper parameter settings can lead to over-clustering or under-clustering, which may negatively impact the final clustering results. To address this, this paper introduces an adaptive neighborhood calculation method for point cloud data, dynamically adjusting the neighborhood radius and minimum sample size based on the local distribution of each point. Specifically, the neighborhood radius is determined by calculating the average distance of the n nearest points for each point, and the minimum sample size is determined based on the point density within the neighborhood. This approach enables DBSCAN to perform more adaptively and robustly across diverse datasets and environments.

The approach can be detailed through the following steps:

(1) Calculate the distances to the n nearest neighbors for each point: For each point $p_{i}$ in the point cloud, calculate the distances to all other points in the cloud. The n nearest neighbors are selected based on the smallest distances.

(2) Compute the average distance d: Once the n nearest neighbors are identified for each point, calculate the average of these distances as follows:

(11) $$d=\frac{1}{n}\sum_{i=1}^{n}dsitance(p_{i},p_{j})$$

where distance $\left(p_{i},p_{j}\right)$ is the Euclidean distance between point $p_{i}$ and its j-th nearest neighbor.

(3) Determine the neighborhood radius: Set the neighborhood radius to 2d, ensuring that the radius is large enough to cover the local neighborhood of each point as follows:

(12) eps=2d

(4) Set the minimum sample size: The minimum number of points within this radius (density threshold) is determined as half of n, to balance computational efficiency and the robustness of clustering as follows:

(13) $$minpts=\frac{n}{2}$$

where n is chosen as 8 to maintain computational efficiency while ensuring that the density is sufficiently large to guarantee reliability.

This method dynamically adjusts the parameters based on the local point cloud distribution, allowing for more adaptable and stable clustering in various environments and datasets.

**Figure 3:**
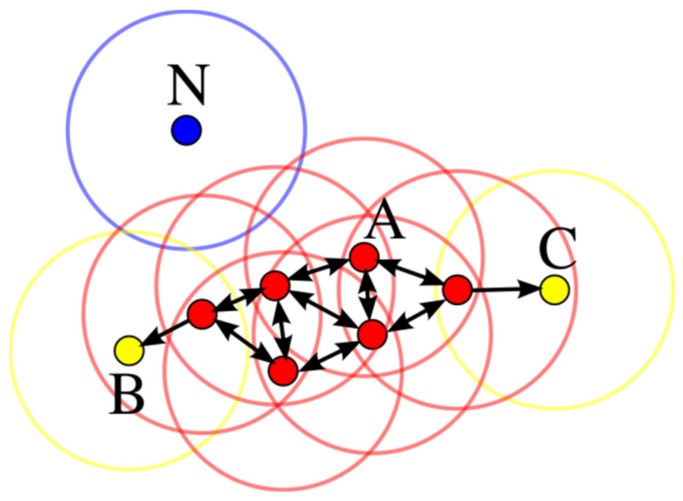
A clustering example of DBSCAN where minpts = 4.

Figure 3 illustrates a clustering example of DBSCAN, where minpts=4. In this figure, point A and other red points are considered core points as their neighborhood (represented by red circles) contains at least 4 points, including themselves. Being mutually reachable, they form a cluster. Although points B and C are not core points, they are reachable through core point A and other core points, making them part of the same cluster. Point N is classified as a noise point since it is neither a core point nor reachable from other points.

For the CSNDT algorithm, the objective is to obtain straight-line point cloud clusters. While the traditional DBSCAN algorithm performs well in clustering point clouds of arbitrary shapes, adjustments are necessary for the CSNDT algorithm to meet its specific requirements. Specifically, a judgment mechanism is proposed during the clustering process of DBSCAN for CSNDT. When a core sample is added to cluster C, this judgment mechanism is triggered, and it calculates the covariance matrix cov(X,Y) and correlation coefficient R for points within C, including the newly added core sample. The corresponding calculation equations are as follows:

(14) $$\mathrm{cov}(X,Y)=E[(X-\mu_{x})(Y-\mu_{y})]$$

(15) $$R=\frac{\mathrm{cov}(X,Y)}{\sigma_{x}\sigma_{y}}$$

(16) $$\mu_{x}=E(X),\;\mu_{y}=E(Y)$$

(17) $$\sigma_{x}=\sqrt{\frac{\sum {\left(X-\mu_{x}\right)}^{2}}{n}},\;\sigma_{y}=\sqrt{\frac{\sum {\left(Y-\mu_{y}\right)}^{2}}{n}}$$

where X,Y denote the *x*-axis and *y*-axis coordinates of the two-dimensional point cloud, respectively, μ represent the means, and σ stand for the standard deviations. The coefficient of correlation, denoted as R, reflects the degree of closeness in correlation between the x and y coordinates of the point cloud. As R increases, it signifies a higher correlation between the *x* and *y* axes, thereby indicating a tendency for the point cloud cluster to be more linear. However, in cases where the point cloud cluster is oriented vertically or horizontally, even if the cluster appears linear, the coefficient of correlation R might still be relatively small. To address this limitation, a secondary criterion is introduced: the standard deviation. This metric assesses the concentration of the point cloud along the *x* or *y* direction, with smaller standard deviation values indicating higher concentration and greater linearity. Consequently, two threshold values have been established to evaluate the suitability of newly incorporated points. The specific rules for assessment are outlined as follows:

(18) $$R>\eta_{R}$$

(19) $$\sigma_{x}<\eta_{\sigma }\;\mathrm{or}\;\sigma_{y}<\eta_{\sigma }$$

where $\eta_{R}$ and $\eta_{\sigma }$ represent the thresholds for the coefficient of correlation and the standard deviation, respectively. When a newly added point satisfies either of these two thresholds, it will be categorized into the same cluster.

### 4.2. Adaptive Generating and Segmenting of Cells

Adaptive grid cells can be generated through linear point cloud clusters. Initially, the mean and covariance of each point cloud cluster C are computed. Based on the size and location of $C_{i}$, initial cells are adaptively generated, determining the boundaries $F_{i}$ of the grid cells and calculating their lengths l. Subsequently, an assessment is conducted to determine whether l exceeds a predefined length threshold $\varepsilon_{l}$. If this is the case, the initial cell is further subdivided into overlapping smaller cells. The mathematical representation of this process is as follows:

(20) $$F_{i}=\{x_{\min},x_{\max},y_{\min},y_{\max}\}$$

(21) $$l=\sqrt{{\left(x_{\max}-x_{\min}\right)}^{2}+{\left(y_{\max}-y_{\min}\right)}^{2}}$$

(22) $$seg=\left\lceil l/\varepsilon_{l}\right\rceil$$

where $x_{\min},\;x_{\max},\;y_{\min},\;y_{\max}$ represent the boundaries of function $F_{i}$, which are influenced by the maximum and minimum values of coordinates along the *x* and *y* axes within the point cloud cluster $C_{i}$.

**Figure 4:**
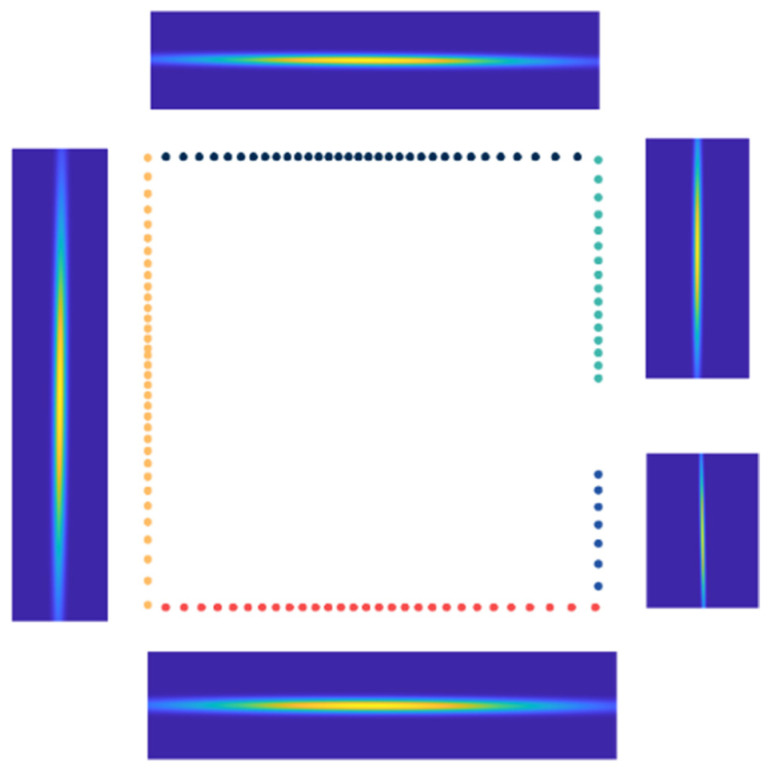
CSNDT map.

**Figure 5:**
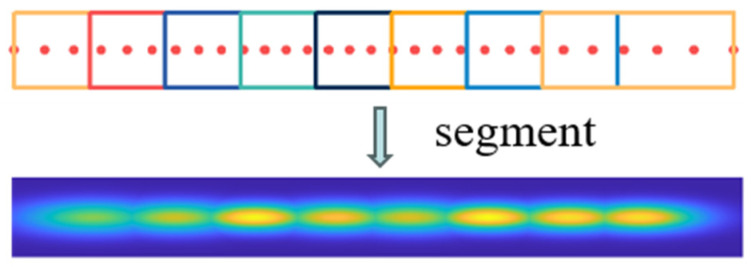
Illustration of where the initial cells with lengths exceeding the threshold are segmented.

During the initial cell subdivision process, as the point cloud clusters are segmented into straight lines, the width of the original cell boundaries tends to be narrow. To enhance the likelihood of mapping points falling within the cells and to broaden the scope of point cloud registration, these boundaries are expanded. Regarding the segmentation of the initial cells, it is used in the second optimization-solving phase, with initial values derived from the first optimization-solving phase. When more precise initial values are available, we employ either the boundaries $F_{i}$ or slightly expanded boundaries. By employing the L-DBSCAN algorithm for point cloud clustering and segmentation, we attain a CSNDT map as depicted in Figure 4. Distinct point cloud clusters are represented with varying colors, revealing the segmentation of the point cloud into five straight clusters with adaptive cell subdivision. In contrast to conventional NDT algorithms, the CSNDT approach employs a reduced count of grid cells, significantly mitigating storage space requirements and potential time overhead in subsequent optimization-solving phases. Furthermore, the probability density within the CSNDT map is more densely concentrated within the central map region, thereby augmenting the accuracy of mapping points precisely aligned with their respective NDT maps. These enhancements collectively contribute to elevating the precision and robustness of point cloud registration. Figure 5 illustrates the situation where the initial cells with lengths exceeding the threshold are segmented.

The initialized transformation matrix should be applied to each point $x_{i}$ to acquire its corresponding mapped point $x_{i}^{\prime }$. Based on the generation of initial cells, the associated Gaussian distribution grid cell can be determined for $x_{i}^{\prime }$ in order for the first optimization-solving iteration to proceed. The outcomes of the first optimization-solving iteration can be transmitted to the second iteration, followed by determining the pertinent Gaussian distribution grid cell based on the segmentation of initial cells and continuing the optimization process.

Algorithm 1 provides a concise overview of the workflow for the CSNDT algorithm. The input consists of the reference point cloud Y, utilized to construct the NDT map, and the current point cloud X, which necessitates matching. The algorithm’s output is the transformation matrix p that facilitates the successful alignment of the two-point clouds.
**Algorithm 1.** CSNDTInput:   X: Current scan   Y: Reference scanOutput:   p: Transform parameter 1:{Initialization:} 2:$Y=\{C_{1},\;C_{2},\;\ldots ,\;C_{n}\}$ ← L-DBSCAN 3:For all Point cloud cluster $C_{i}\in Y$ do 4:  $C_{i}=\{m_{1},\ldots ,m_{m}\}\;\leftarrow$ all points in $C_{i}$ 5:  $\mu_{j}\leftarrow \frac{1}{n}\sum_{i=1}^{m}m_{j}$ $m_{j}\in C_{i}$ 6:  $\sum_{j}\leftarrow \frac{1}{n}\sum_{j=1}^{m}(m_{j}-\mu_{j}){\left(m_{j}-\mu_{j}\right)}^{T}$ 7:  $l=\max(x_{\max}-x_{\min},\;y_{\max}-y_{\min})$ 8:  If seg>1 do segmentation 9:    For all small Point cloud cluster $C_{i}^{s}\in C_{i}$ do 10:      $C_{i}^{s}=\{m_{1},\ldots ,m_{m}\}\;\leftarrow$ all points in $C_{i}^{s}$ 11:      $\mu_{j}^{s}\leftarrow \frac{1}{n}\sum_{i=1}^{m}m_{j}$ $m_{j}\in C_{i}$ 12:      $\sum_{j}^{s}\leftarrow \frac{1}{n}\sum_{j=1}^{m}(m_{j}-\mu_{j}^{s}){\left(m_{j}-\mu_{j}^{s}\right)}^{T}$ 13:    End for 14:  End if 15:End for 16:{First Registration:} 17:While not converged do 18:  $score\leftarrow 0,\;g\leftarrow 0,\;H\leftarrow 0,\;p_{1}\leftarrow 0$ 19:  For all points $x_{i}\in X$ do 20:    Find the cell $F_{i}$ that contains $x_{i}^{\prime }$ 21:    $score\leftarrow score+p(x_{i}^{\prime })$ 22:    Update g, H 23:  End for 24:  Solve $H\Delta p_{1}=-g$ 25:  $p_{1}\leftarrow p_{1}+\Delta p_{1}$ 26:End while 27:{Second Registration:} 28:While not converged do 29:  $score\leftarrow 0,\;g\leftarrow 0,\;H\leftarrow 0,\;p_{2}\leftarrow p_{1}$ 30:  For all points $x_{i}\in X$ do 31:    Find the cell $F_{i}^{s}$ that contains $x_{i}^{\prime }$ 32:    $score\leftarrow score+p(x_{i}^{\prime })$ 33:    Update g, H 34:  End for 35:  Solve $H\Delta p_{2}=-g$ 36:  $p_{2}\leftarrow p_{2}+\Delta p_{2}$ 37:End while

## 5. Experiments and Results

To evaluate the performance of the proposed algorithm, experiments were conducted in two distinct environments: one utilizing the “lidarScans.mat” lidar dataset provided by Matlab 2021b, and the other employing point cloud data generated within a simulated environment. The real-world dataset used in Experiment I comprises a range of indoor structures, including walls, doors, and smaller items like furniture. In contrast, the simulated dataset employed in Experiment II features a more varied category distribution, encompassing linear obstacles, curved surfaces, and dense point clusters that represent complex features. This broader diversity enables a comprehensive evaluation of the algorithm’s adaptability to diverse point cloud characteristics. All algorithms were implemented in Matlab and executed on a Windows operating system. The standard NDT algorithm source code was furnished by Matlab. Optimization-based methods were equipped with termination criteria, halting the optimization process when the gradient norm or step size norm fell below 10^−6^.

### 5.1. Experiment I

**Figure 6:**
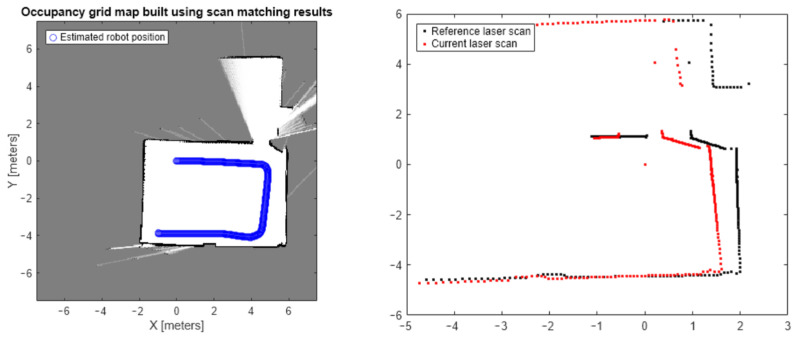
The collection environment of the point cloud dataset.

The experiment employed the “lidarScans.mat” lidar scanning dataset included with Matlab, which consists of real-world two-dimensional lidar scanning data collected indoors. As the dataset does not provide actual lidar positions, random point cloud data were chosen from the dataset, and a transformation matrix was applied to simulate the transformation. The collection environment of the point cloud dataset and the adjacent frame point clouds are illustrated in Figure 6, where the blue line denotes the trajectory of the robot’s movement. The dataset comprises two-dimensional point cloud data, and no preprocessing steps, such as filtering or transformation, were applied prior to the analysis. This unprocessed dataset reflects the raw conditions encountered during data acquisition, ensuring that the results directly demonstrate the algorithm’s performance under practical scenarios. The original point cloud served as the reference, and the transformed point cloud was the current point cloud. These two-point clouds were then input to the matching algorithm to obtain the matching results, and the Root Mean Square Error (RMSE) of the corresponding points was calculated as an assessment criterion for matching effectiveness. Detailed information on key parameters, such as clustering thresholds and segmentation strategies, is provided to guide reproducibility. Given that the reference and current point clouds have identical distributions in this particular scenario, ICP registration holds a noticeable advantage. Nonetheless, it is highly improbable to encounter such circumstances in real-world scenarios. Consequently, this experiment solely aimed to compare performance with the NDT algorithm, while a performance comparison with the ICP algorithm will be conducted in the subsequent experiment.

To assess the algorithm’s robustness, runtime, and accuracy, 300 frames of point cloud data were randomly selected and subjected to various rotation and translation matrices. Translations were applied along the *X* and *Y* axes, while rotations were performed around the origin to test the algorithm’s performance under different rotation and translation conditions. Translation distances ranged from 0.1 m to 2.5 m, and rotation angles ranged from 0.1 radians to 0.8 radians. For each combination of rotation and translation matrix, the number of successful point cloud matches, and the average runtime were recorded. A threshold of ε=0.01 m was set, and if the calculated RMSE after point cloud registration exceeded ε, the match was considered unsuccessful.

**Figure 7:**
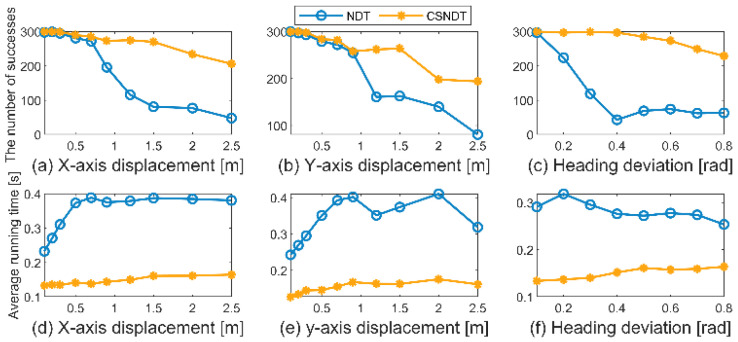
Comparisons of number of successful matches and average runtime.

The experimental outcomes are illustrated in Figure 7. As shown in Figure 7a–c, when the offset distance along the *X* and *Y* axes is less than 0.7 m, there is little difference in the number of successful matches between the CSNDT and NDT algorithms. However, beyond an offset distance of 0.7 m, the number of successful matches for the NDT algorithm declines rapidly, while the CSNDT algorithm maintains a steady success rate with only a minor decrease. Regarding rotation angles, the success rate of the NDT algorithm decreases significantly as the rotation angle increases. At a rotation angle of 0.4 radians, the NDT algorithm achieves a success rate of only 14.3%, with 43 successful matches. In contrast, the CSNDT algorithm achieves 297 successful matches, resulting in a success rate of 99%. These findings demonstrate that the CSNDT algorithm notably enhances robustness compared to the NDT algorithm, particularly in terms of adaptability to rotation scenarios.

In terms of computational efficiency, Figure 7d–f compare the runtime of the algorithms under different rotation and translation matrices. The runtime of the NDT algorithm fluctuates between 0.233 s and 0.42 s, with an average of 0.327 s. In contrast, the runtime of the CSNDT algorithm ranges from 0.127 s to 0.176 s, with an average of 0.150 s, and exhibits less variability. As the rotation and translation matrices increase, the runtime of the CSNDT algorithm gradually increases, but it remains more efficient than the NDT algorithm. The results indicate that the CSNDT algorithm is 0.177 s faster than the NDT algorithm, representing a 54.1% improvement in efficiency.

**Figure 8:**
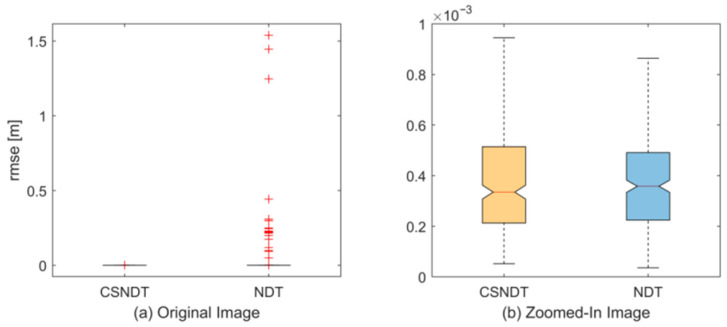
Matching error comparisons.

To evaluate the algorithm’s accuracy, this study selected a rotation and translation matrix *p* = [0.1 0.1 0.1] to minimize the impact of matching failures on point cloud alignment precision. The experimental results are presented in Figure 8. While the precision discrepancy between the two algorithms is minimal, the CSNDT algorithm demonstrates a lower median value and fewer outliers, indicating a slight improvement in matching accuracy.

### 5.2. Experiment II

**Figure 9:**
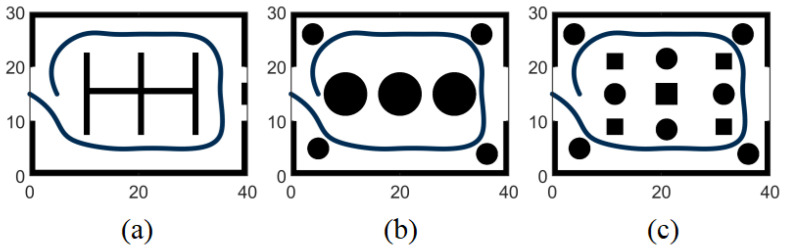
Environmental map. The deep blue points represent the actual trajectory points of the vehicle. Subfigures (**a**–**c**) each illustrate environment maps generated with different levels of complexity.

In this experiment, a simulated map, as shown in Figure 9, was initially constructed along with a realistic vehicle trajectory. To thoroughly assess the algorithm’s reliability, three environment maps of varying complexity were generated by simulating a warehouse setting. The first scenario consisted solely of wall segments and shelves without any additional obstacles, as depicted in Figure 9a. The second scenario included wall segments and circular obstacles, as illustrated in Figure 9b. The third scenario presented a combination of wall segments, circular obstacles, and rectangular obstacles, as illustrated in Figure 9c. In the simpler environment, the obstacles shared similar visual characteristics, allowing the lidar sensor to clearly outline their contours. However, minimal variations were observed in the data from frame to frame, leading to repetitive data representation. In the more complex environment, obstacles featured distinct point, line, and surface attributes. Although the lidar sensor captured a more comprehensive range of obstacle information, it struggled to precisely outline the contours of the obstacles. By conducting comparative experiments in these three distinct environments of varying complexity, the performance of the algorithm could be effectively validated.

The actual trajectory $Tr_{true}$ comprises 400 trajectory points $Tr_{true}^{i}$, with each point recording the vehicle’s true position and heading angle. The lidar data obtained at each trajectory point are used as the dataset for evaluation. To comprehensively evaluate the algorithm’s robustness, all algorithms were initialized with zero translation and zero rotation parameters. In practical applications, initial pose estimates are typically provided to the algorithm using additional sensors or data generated from motion models, thereby reducing the number of iterations required for convergence.

**Figure 10:**
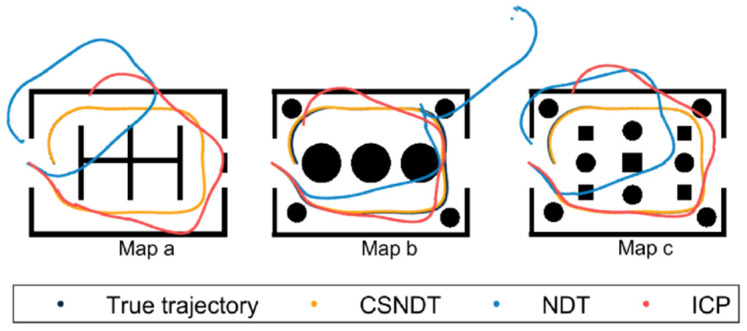
Comparison of trajectories generated by different methods.

The algorithm takes the data from the *i*-th and (*i* + 1)-th frames as input to obtain the rigid transformation matrix between them. Subsequently, according to the transformation matrix, the vehicle’s trajectory, referred to as the generated trajectory $Tr_{generate}$, is calculated. The error of the generated trajectory relative to the true trajectory accumulates over time. As illustrated in Figure 10, different colors represent trajectories generated by different algorithms. The orange trajectory points, generated by the CSNDT algorithm, closely mirror the true trajectory across all three environments. Particularly in Environment (a), the generated trajectory almost perfectly aligns with the true trajectory. In Environments (b) and (c), the generated trajectory exhibits slight deviations from the true trajectory. The light blue points represent trajectory points generated by NDT, which results in considerable deviations across all three environments. In Environment (c), the NDT algorithm fails to match the 322nd trajectory point, leading to a significant deviation from the true trajectory at subsequent points. The pale pink points correspond to the trajectory generated by the ICP algorithm, which also demonstrates significant offsets from the true trajectory. The experimental results indicate that, compared to NDT and ICP, the proposed CSNDT method demonstrates superior matching performance across environments of varying complexity. This advantage is particularly evident in settings dominated by linear obstacles, where the CSNDT method achieves the highest accuracy in trajectory alignment.

To deliver a more accurate depiction of the matching performance across various algorithms, cumulative errors were eliminated and the errors relative to $Tr_{true}$ after each individual match were assessed. Therefore, $Tr_{generate}^{\prime }$ was computed based on $Tr_{true}$ using the obtained transformation matrix between the *i*-th and (*i* + 1)-th frames, stated as follows:

(23) $$Tr_{generate}^{\prime i+1}=Tr_{true}^{i}\times p$$

**Figure 11:**
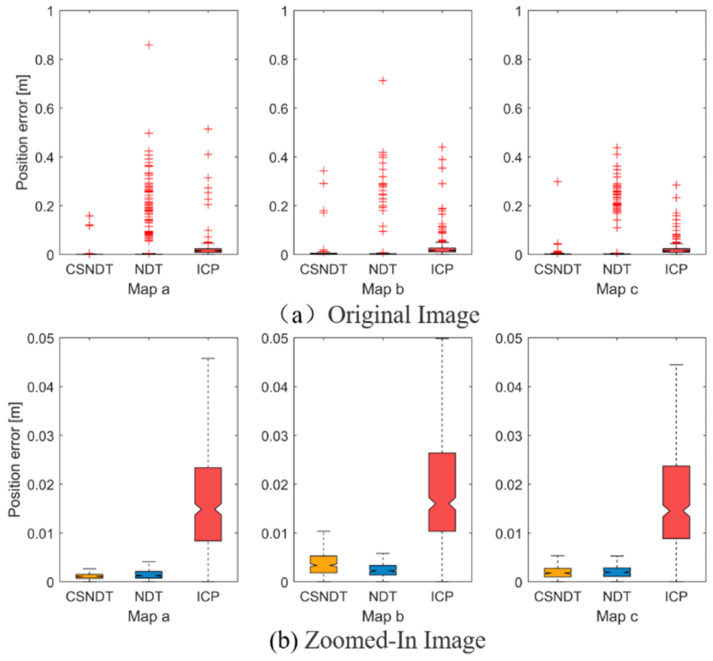
Position error comparisons.

**Figure 12:**
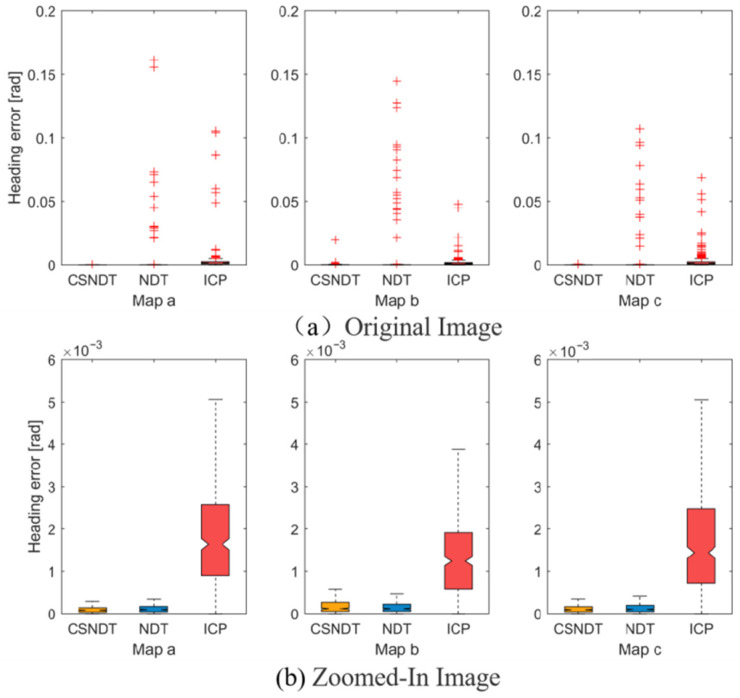
Angular error comparisons.

Figure 11 and Figure 12 display the position error and heading angle error of the three algorithms, where the original image depicts the position and quantity of these outliers. The location and quantity of outliers, to a certain extent, reflect the frequency and extent of algorithmic matching failures. Experimental findings reveal that CSNDT consistently produces significantly fewer outliers and smaller deviations across all three environments compared to NDT and ICP. Figure 11b and Figure 12b represent an amplified view of the data after removing the outliers, highlighting the error magnitude for successful matches. It can be observed from the figures that CSNDT achieves the highest accuracy in Environment (a), performs slightly less accurately than NDT in Environment (b), and shows comparable performance with NDT in Environment (c). These results indicate that the proposed algorithm excels in linear scenarios, maintaining a clear advantage in environments with pronounced linear features. While its accuracy slightly declines when encountering curvilinear obstacles in Environment (c), CSNDT continues to exhibit a robust and reliable performance.

## 6. Discussion

The improved DBSCAN clustering algorithm demonstrates significant advantages when processing reference point clouds, effectively segmenting them into point cloud clusters with clearly defined linear features. However, its clustering performance is influenced by multiple key parameters, including the neighborhood radius (Epsilon), the minimum sample size (MinPts), the correlation coefficient threshold, and the standard deviation threshold. Each parameter has a significant impact on the clustering outcome. Due to the diversity of LiDAR data and the differences in point cloud features across various application scenarios, these parameters cannot be universally applied and must be adjusted flexibly based on the specific operational conditions of the LiDAR (such as scanning angle and resolution) as well as the actual environment in which the robot operates (such as outdoor, indoor, or dynamic scenes) to achieve optimal clustering results.

Furthermore, the method proposed in this paper demonstrates remarkable robustness, enabling the segmentation of point cloud data into linear point cloud clusters of varying sizes. This segmentation capability is particularly suitable for extracting linear features in structured environments, such as indoor corridors and urban streets. In these settings, elements like walls, curbs, and buildings exhibit distinct linear boundaries that can be distinctly segmented by this algorithm. Moreover, the method also shows strong adaptability to obstacles with slight curvature, such as gently curved walls or rounded edges, because small curvature curves can be approximated by combining multiple straight lines, forming continuous linear clusters.

However, in more complex environments, particularly those with obstacles characterized by significant curvature, the limitations of the method become apparent. For example, objects like thin rods that exhibit significant curvature may be incorrectly identified as isolated noise, resulting in the loss of important information. This occurs because the nature of linear fitting limits the effective handling of non-linear structures with large curvature. Consequently, the assessment of linearity in point cloud clusters may erase the features of these large curvature objects. To overcome this challenge, it is essential to develop advanced curve clustering and segmentation algorithms capable of effectively handling large curvature features. Additionally, incorporating more adaptable point-mapping scoring mechanisms can facilitate precise analysis and processing of point cloud data across varied environments. These improvements would not only enhance the accuracy of point cloud segmentation but also significantly bolster the algorithm’s adaptability to complex and dynamic scenarios.

Currently, the proposed improved algorithm has only been tested and validated in a two-dimensional point cloud environment, leaving its applicability in three-dimensional point cloud scenarios yet to be thoroughly examined. While point cloud processing in two-dimensional settings is relatively straightforward, the transition to three-dimensional environments introduces significantly greater complexity due to the richer spatial information and intricate structural features present in 3D data. Expanding the algorithm’s capabilities to three-dimensional environments represents a challenging yet highly promising avenue for future research. This endeavor should prioritize optimizing the algorithm for 3D point cloud processing, enabling the effective segmentation of planar and surface features within three-dimensional space. Furthermore, the development of adaptive voxel generation techniques will be crucial to accommodate the additional dimensionality and complexity. Such advancements would offer more precise and efficient solutions, particularly in applications like robotic localization and environmental perception, paving the way for enhanced performance in real-world scenarios.

## 7. Conclusions

This paper proposes a cell division method based on point cloud clustering and segmentation to address challenges in the initialization phase of the NDT algorithm, which can result in the loss of local point cloud feature information and mapping errors. An improved DBSCAN clustering algorithm is employed to segment the point cloud into linear clusters. Each point cloud cluster is adaptively divided into grid cells, and when the length of a grid cell reaches a threshold, it is further divided into overlapping smaller grid cells. Two optimization processes are then carried out: the first optimization enhances the algorithm’s robustness and provides an initial value for the second optimization, which aims to improve matching accuracy. Experimental validations conducted across environments of varying complexity demonstrate that the proposed CSNDT algorithm significantly outperforms traditional methods in robustness, matching efficiency, and accuracy, proving effective in both simple and intricate environmental scenarios.

## Data Availability

The data that support the findings of this study are available from the author, Y.T., upon request.
